# Gestural Embodiment of Intensifiers in Iconic, Metaphoric, and Beat Gestures

**DOI:** 10.3390/bs13020174

**Published:** 2023-02-15

**Authors:** Omid Khatin-Zadeh, Danyal Farsani, Jiehui Hu, Zahra Eskandari, Hassan Banaruee

**Affiliations:** 1 School of Foreign Languages, University of Electronic Science and Technology of China, Chengdu 610054, China; 2 Department of Teacher Education, Norwegian University of Science and Technology, 7491 Trondheim, Norway; 3 Department of English, Chabahar Maritime University, Chabahar 99717-56499, Iran; 4 Department of English, American, and Celtic Studies, University of Bonn, 53115 Bonn, Germany

**Keywords:** embodiment, intensifier, iconic gesture, literal sentence, metaphoric gesture, metaphoric sentence

## Abstract

This study aimed to examine the gestural embodiment of intensifiers in iconic and metaphoric gestures when these words are used with literal and metaphoric statements. We asked a group of Persian native speakers to listen to and then retell a set of Persian stories. In these stories, a number of intensifiers were used with literal and metaphoric sentences. The results showed that when an intensifier was used with a literal sentence, there was a higher probability of using an iconic or beat gesture than when there was no intensifier in the sentence. Also, when an intensifier was used with a metaphoric sentence, there was a higher probability of using a metaphoric or beat gesture than when the sentence contained no intensifier. These results suggested that an intensifier in a literal or metaphoric sentence can strengthen the mental simulation and the embodiment of objects, ideas, or situations. When an intensifier is used with a literal or metaphoric sentence, the strength of activation in the premotor areas may be amplified and spread to motor areas. In contrast, when no such intensifier is used in a literal or metaphoric sentence, there is a higher probability of simulation in premotor areas without spreading to the primary motor areas. The production of an internal force and expressing emphasis are two other possibilities that may explain the higher use of gestures with intensifiers.

## 1. Introduction

Embodied language cognition has been one of the widely debated topics in cognitive science in recent years. Embodied language cognition refers to the reactivation of sensorimotor experiences when people use language to talk about something or an experience (e.g., [1,2,3,4]). According to the main idea of embodiment theories, the same sensorimotor processes involved in the actual perception of an object or an experience are reactivated when people talk about that object or experience (e.g., [5,6,7,8], for reviews, see [9,10]). The reactivation of sensorimotor experiences can happen in various modalities; visual, auditory, haptic, gustatory, olfactory, and motoric experiences involved in perceiving an object or having an experience can be reactivated when people talk about it. This means that the experience is mentally simulated, and the same sensorimotor networks used to perceive the object or experience are reactivated.

Generally, embodied language cognition can be divided into two areas of embodied literal language cognition and embodied metaphoric language cognition. When people use literal language, the words directly refer to their referents. For example, *the table is made of wood* is a literal sentence as all words have been used in their literal senses in this sentence. According to embodied literal language cognition (e.g., [4,7,8]), when this sentence is used, the same sensorimotor experiences and neural processes involved in perceiving a *table*, which are primarily visual and haptic, are reactivated. To take another example, when people talk about cinnamon, the sensory experiences involved in perceiving *cinnamon* (visual, olfactory, and gustatory) are activated [11,12]. According to embodied metaphoric language cognition, the reactivation of sensorimotor experiences takes place even when people use metaphoric language [13]. It has been argued that processing a metaphoric sentence that describes something (target of the metaphor) in terms of an action (base of the metaphor) involves simulating the metaphorical action [13]. For example, in the metaphorical phrase *grasp an idea*, understanding something (target of the metaphor) is metaphorically described in terms of grasping an object (base of the metaphor). From the perspective of embodied metaphoric language cognition, while processing this metaphor, the action of grasping a physical object is simulated in the mind. Here, the action of grasping is a metaphorical action, but it is simulated like a real action. The key assumption of embodied metaphoric language cognition is that the same sensorimotor networks that are engaged to perform or perceive the base of a metaphor (e.g., grasping an object) are recruited to process the target of the metaphor and the whole metaphorical statement. Embodied metaphorical processing has been supported by the findings of some behavioral experiments (e.g., [14,15,16,17]) and neuroimaging studies (e.g., [18,19,20]).

The actions, objects, or events that are described by literal or metaphoric sentences can be simulated mentally or physically. Mental simulation is entirely an internal process that involves only the activation of neural networks. Interestingly, there is some neuroimaging evidence suggesting that the same neural networks that are engaged in the actual perception of an object or experience are also engaged in mental imagery or mental simulation of that object or experience (e.g., [21,22,23,24]). For example, when an experience has been perceived through vision, the process of mental simulation of that experience involves the activation of the visual system (e.g., [25]). On the other hand, physical simulation is an external process that can take place in gestures. For example, the action of pushing an object can be simulated by a pushing gesture. While the mental simulation of pushing an object takes place at a mental level that cannot be perceived by an observer, the gestural simulation takes place at a physical level that can be perceived by an external observer.

Studies on co-speech gestures show that people mentally simulate actions, concepts, and events during talking [26,27]. Such mental and gestural simulations accompany both literal and metaphoric statements. For example, when people use the literal sentence *I pushed the car*, they may use a pushing gesture. This use of this gesture indicates that the action described by the literal sentence is mentally and physically simulated. Interestingly, people may use a pushing gesture when they produce the metaphoric sentence *he pushed his idea*. Here, the action of pushing is not a real action but a metaphorical one. This suggests that even metaphorical actions are mentally simulated and appear in the form of co-speech gestures when people use metaphoric language [26,27,28,29,30]. Based on the function and the information that is provided by gestures and also types of co-occurring sentences, gestures can be classified into different categories. In the following section, we take a look at one of the most well-known classifications of gestures. Then, we describe the methodology of our study that aimed to investigate how intensifiers (very, too, so, etc.) are embodied in gestures when they are used with literal or metaphoric sentences.

## 2. Various Types of Gestures

McNeill’s typology [31] is one of the most useful and well-known classifications of gestures, which has been particularly used by researchers studying the cognitive aspects of gestures. Based on this typology, gestures are classified into four main classes: (1) pointing (deictic) gestures, (2) iconic gestures, (3) metaphoric gestures, and (4) beat gestures. Pointing gestures are totally dependent on the physical context of the conversation and are used to refer to objects or locations in the area of conversation. A pointing gesture often takes the shape of an extended index finger and shows the direction that leads to the object or the place it refers to. In addition to the index finger, other fingers or the entire hand may also be used as a pointing gesture. However, the index finger is the most common finger used to produce pointing gestures. An iconic gesture presents an illustration of the shape of an object or a physical feature of an event by the shape of the hand or the trace of hand movements. An iconic gesture has an iconic and meaningful relationship with the shape or the meaning of the object it refers to. For example, illustrating a circle by the trace of a gesture for referring to the circular shape of an object is an iconic gesture. A metaphoric gesture refers to the metaphorical meaning of an expression and illustrates the base of the metaphor to refer to its target. For example, a grasping gesture that is used with the metaphoric sentence *I grasped the idea* is a metaphoric gesture. In this example, *understanding an idea* (target of the metaphor) is understood in terms of *grasping an object* (base of the metaphor), and a grasping gesture is used to illustrate the base of the metaphor. Here, the metaphoric gesture illustrates the metaphorical action of grasping. The beat gesture does not have any meaningful relationship with the accompanying speech. It does not express any semantic information. It is just produced in some kind of coordination with the prosody of speech. Iconic and metaphoric gestures are put in one category and are called representational gestures.

Among the four types of gestures in McNeill’s typology, iconic and metaphoric gestures have the closest relationship with the meanings of words that are used with them. In the following section, we review some works that have discussed these two types of gestures. Then, we describe the methodology of our study that aimed to investigate the embodiment of intensifiers in these types of gestures.

## 3. Iconic and Metaphoric Gestures

In order to describe the processes and mental mechanisms of gesture production and comprehension, several theories have been suggested, such as the sketch model [32], the lexical gesture process model [33], the interface model [34], the growth point theory [35,36], and the gesture-in-learning-and-development framework [26]. The gesture-as-simulated action [27] is one of the latest theories that have been successful in explaining the mental mechanisms of gesture production. This theory holds that gestures are the result of mental simulations of perceptual and motoric properties. Based on this theory, when a literal sentence is used to describe spatial and motoric properties of an idea, or when a metaphoric sentence is used to describe an idea in terms of spatial and motoric properties, a gesture may be produced, regardless of whether the idea is literally or metaphorically described [27]. Iconic gestures are the result of simulating real spatial and motoric features, while metaphoric gestures are the result of metaphoric simulations of ideas in terms of spatial and motoric concepts.

In our recent study on the use of metaphoric and beat gestures with metaphors [37], metaphors were categorized into four types (motion-based, static space-based, static object-based, and static event-based) and the use of metaphoric and beat gestures with each type of metaphor in retelling stories was examined. The results showed that static space-based metaphors were accompanied by the highest number of metaphoric gestures, while static event-based metaphors were accompanied by the highest number of beat gestures. Since the classification of metaphors was based on the spatial and motoric properties of the base of the metaphors, such results suggest that the use of metaphoric gestures and beat gestures with metaphors is largely on the spatial and motoric properties of the base of the metaphors. In an extension of the previous study [38], the same idea that had been used to categorize metaphors was also used to categorize literal sentences into four types. Then, the use of metaphoric and iconic gestures with the four types of metaphorical sentences and the four types of literal sentences in retelling stories was examined. The results showed that event-based metaphors were used with the smallest number of metaphoric gestures, and event-based literal statements were used with the smallest number of iconic gestures, again suggesting that the use of gesture is highly dependent on spatial and motoric properties of concepts that are described in the accompanying speech.

In one study that was investigated to examine the impact of metaphoric gestures on metaphor comprehension [39], participants performed a metaphoric gesture or imagined a metaphoric gesture that illustrated the base concept of a subsequent metaphor. The results showed that performing or even imagining a metaphoric gesture could facilitate the process of understanding the subsequent metaphor. A related recent study [15] examined a group of participants’ comprehension of metaphors in congruent gesture-prime conditions, opposite gesture-prime conditions, and no-prime conditions. In congruent gesture-prime conditions, each metaphor was preceded by a metaphoric gesture that was congruent with the gestural representation of the schema of the subsequent metaphor. In opposite gesture-prime conditions, each metaphor was preceded by a gesture that was opposite to the gestural representation of the schema of the subsequent metaphor. The results showed that participants of congruent gesture-prime conditions performed better than the other two groups in comprehending metaphors.

Results of the reviewed studies support the idea that literal and metaphoric actions are mentally simulated (embodied) and can appear in co-speech iconic and metaphoric gestures. In this study, we aimed to investigate the gestural embodiment of intensifiers in iconic and metaphoric gestures when these words are used with literal and metaphoric statements. To this aim, we asked a group of Persian native speakers to listen to and then retell a set of Persian stories. In these stories, a number of intensifiers were used with literal and metaphorical sentences. We examined the reproductions of these stories by the participants to find out how intensifiers are realized in iconic and metaphoric gestures.

## 4. Method

### 4.1. Participants

We randomly selected a group of thirty students from language learners at Bartar Language Academy. The participants were selected from a larger heterogeneous group of students who were available for the study. This group consisted of 18 females and 12 males. They were between 17 and 28 years old. However, we did not consider gender and age as variables in our study. All participants were native Persian speakers.

### 4.2. Materials

We used four audio stories in the study. Each story included 250–300 words and was presented in around three minutes. The stories were told in Persian. The first story was about the early hardships and difficult life of a neglected poor man who later became a successful, rich man. The second story was about a simple worker who later became a top manager through hard work and extraordinary talent. The third story was about the positive results of an apparently bad event for the first character of the story. The fourth story was about the value of a kind heart. In each story, six intensifiers were used. The English translations of these intensifiers were as follows: very, too, so, really, rather, completely. Each intensifier was used twice in each story, once with a literal sentence and another with a metaphoric sentence. Therefore, each story contained twelve intensifiers. The order of literal and metaphoric sentences in the stories was random. The English translations of literal and metaphoric sentences are given in Appendix A.

### 4.3. Procedure

Before conducting the main part of the study, participants were trained in a session designed to familiarize them with the procedure and the conditions of the study. In this training session, each participant listened to a sample story and retold it in front of a camera. In order to avoid any effect on the performance of the participants, the purpose of the study was not revealed to the participants. In addition to this training session, just before conducting the main study, participants were provided with oral instructions. First, they listened to the first audio story. The story was presented to the participants by a speaker. A camera had been installed two meters away from the participants to record gestures while retelling the stories. While listening to the stories and retelling them, participants were in a standing position. The story was played twice. After listening to the story for the second time, the participants retold the story. They were given three minutes to retell the story. The distance between the participants and the camera made the recording of the gestures produced during the retelling of the story possible. The same procedure was used for the other three stories. These stories were presented to all participants in the same order.

### 4.4. Data Analysis

The video recordings were analyzed to obtain the number of literal and metaphoric sentences that had been used while retelling the stories. First, the number of literal sentences used with intensifiers and the number of literal sentences that had been used without intensifiers were obtained. For each group of these literal sentences (with or without an intensifier), the number of literal sentences used with iconic gestures, beat gestures, or without a gesture was obtained. A similar procedure was used for metaphoric sentences. The number of metaphoric sentences used with and without intensifiers was obtained. For each group of these metaphoric sentences (with intensifier or without intensifier), the numbers of metaphoric sentences used with metaphoric gestures, beat gestures, or without gestures were obtained. Also, a Chi-square test was used to examine the association between using intensifiers in sentences and using gestures with the sentences. This analysis was done for both literal and metaphoric sentences separately. The coding of gestures was done by two coders who were not involved in the study. This was done to avoid any kind of bias in the process of coding gestures. The inter-coder reliability was calculated by calculating the Kappa coefficient to make sure that the process of coding gestures had an acceptable level of reliability. In those few cases where the coding of the two coders was not consistent, one of the researchers of the study made the final judgment. These analyses had two aims. The first aim was to examine the co-occurrence of intensifiers with iconic gestures, beat gestures, and no-gesture when literal sentences were used. The second aim was to examine the co-occurrence of intensifiers with metaphoric gestures, beat gestures, and no-gesture when metaphoric sentences were used.

## 5. Results

First, the inter-coder consistency in the coding of data was calculated by obtaining the Kappa coefficient. This coefficient was 0.91, which is a high level of reliability. This was an acceptable level of reliability in the coding of the data. As mentioned, in those cases where the two coders had made different judgments on the type of gestures, one of the researchers of the study made the final judgment. The results of the study have been summarized in Table 1 and Table 2. The second row in Table 1 shows the proportion of cases in that intensifiers co-occurred with iconic gestures, beat gestures, or no gestures when literal sentences were used by the participants. Results of the Chi-square test showed a significant association between using intensifiers in literal sentences and using gestures (χ^2^ = 47.64, *p*<0.00001). These values show that when an intensifier is used with a literal sentence, there is a higher possibility of using an iconic or beat gesture than when the sentence is used without an intensifier.

The second row in Table 2 shows the proportion of cases in that intensifiers co-occurred with metaphoric gestures, beat gestures, or no-gesture when metaphoric sentences were used by the participants. Results of the Chi-square test showed a significant association between using intensifiers in literal sentences and using gestures (χ^2^ = 56.44, *p*< 0.00001). These values show that when an intensifier is used with a metaphoric sentence, there is a higher possibility of using a metaphoric or beat gesture than when the metaphor is used without an intensifier.

A comparison between the results presented in Table 1 and Table 2 reveals that when metaphoric or literal sentences are used with intensifiers, the pattern of co-occurrence of intensifiers and gestures is similar. That is, when intensifiers are used with metaphoric or literal sentences, there is a higher probability of using gestures (iconic or beat gestures with literal sentences and metaphoric or beat gestures with metaphoric sentences) than when no intensifier is used with literal or metaphoric sentences.

## 6. Discussion

As mentioned in the previous section, the results of this study showed that when an intensifier was used with a literal sentence, there was a higher probability of using an iconic or beat gesture with the sentence. Also, when an intensifier was used with a metaphoric sentence, there was a higher probability of using a metaphoric or a beat gesture with the sentence. A question that is raised here is why intensifiers raise the probability of using gestures with literal and metaphoric sentences. To answer this question, we provide three explanations in the following three subsections.

### 6.1. Amplifying the Strength of Simulation and Embodiment

One possible answer to the posed question is that intensifiers strengthen the degree of embodiment of the object, idea, or situation that is described by a literal or metaphoric sentence. As mentioned in reviewing past works, results of some studies have provided evidence showing using literal and metaphorical sentences activates mental imagery or sensorimotor mental simulation of the object, idea, or situation that is described by that sentence (e.g., [4,6,11,12,21,22,23,24,25]). Therefore, when these sentences are used with intensifiers, mental simulation and the embodiment of an object, idea, or situation can be strengthened. This strengthening of mental simulation and embodiment prepares the ground for the transformation of simulation and embodiment from a mental realization to a physical gestural realization. In other words, when an intensifier is used with a literal or metaphorical sentence, the process of mental simulation and the embodiment of the object, idea, or situation is strengthened and is realized in the form of gestures. This means that intensifiers can function as an *embodying amplifier*. These embodying amplifiers can support the process of transforming a fully mental simulation or embodiment of an object, idea, or situation into a physical one realized in the form of gestures.

Intensifiers do not have much semantic content when they stand alone. Their meanings are largely dependent on the meanings of other words in the sentence. For instance, in the sentence *he was fighting against a very high mountain of challenges*, the meaning of the intensifier (very) is highly dependent on the meanings of the adjective (high) and the noun (mountain) that have been used after it. Therefore, embodied realization of the intensifier is one part of the embodiment of the words that have been used after it in the sentence. In fact, the intensifier strengthens the process of mental imagery and the embodiment of a high mountain. Here, the intensifier is embodied as the degree of the height of a mountain. Intensifiers have dynamic meanings. They can refer to the degree of height, weight, speed, and many other things. Depending on the meanings of words used in a sentence, an intensifier used in that sentence can amplify the meanings of other words and strengthen the process of embodiment from a fully mental realization to a physical realization in the form of gestures.

The important point about intensifiers is that they do not change the semantic content of the sentence. For example, the meaning of the sentence *the speed of changes was high* does not differ significantly from the meaning of the sentence *the speed of changes was very high.* The only difference in the semantic contents of these two sentences is in the degree of the speed of an event. Since an intensifier (very) has been used in the second sentence, the process of the metaphorical embodiment of the movement of an object (the base of the metaphor is the speedy movement of an object) is strengthened. Therefore, the probability of using a gesture to describe this high speed is increased.

From the perspective of gesture-as-simulated-action theory [26,27], gestures occur when the strength of activation in the premotor areas exceeds a certain threshold and spreads to motor areas. The activation in the premotor areas is triggered by a mental simulation or embodiment in these areas. Therefore, it can be suggested that when a literal or metaphoric sentence is used without an intensifier, there is a higher probability of simulation in premotor areas without spreading to the primary motor areas. In such cases, the strength of activation may not be strong enough to spread to primary motor areas. When an intensifier is used with a literal or metaphoric sentence, the strength of activation in the premotor areas is amplified. In such cases, there is a higher probability of spreading activation from premotor areas to motor areas. When this happens, gestures occur, and the process of embodiment is realized in gestures. In other words, the intensifier functions as a stimulus that causes the spreading of activation from premotor areas to primary motor areas, leading to the production of gestures. This is, in fact, a transformation of a full mental process (simulation and embodiment in the premotor areas) into a physical simulation in gestures.

### 6.2. Triggering an Internal Force in the Body

Since intensifiers are used to express the degree of something (size, weight, speed, etc.), they are often accompanied by the production of an internal force in the body. In other words, the degree of something is embodied as a kind of internal force in the body. This can be realized as muscle intensification. This internal force can be a stimulus for the production of gestures. In other words, the internal force that accompanies an intensifier could prepare the body for the production of a gesture. Furthermore, many Persian intensifiers contain a long, strong vowel. The production of these vowels involves the release of high-pressure air from the lungs. The exit of high-pressure air from the lungs is also accompanied by an extra internal force in the body, which can stimulate the body for the production of a gesture. It should be noted that the use of long, strong vowels is the case not only with Persian intensifiers but also with intensifiers in other languages. For example, most English intensifiers contain long and strong vowels (e.g., too, extremely, completely). The simultaneous production of gesture and release of high-pressure air from the lungs is particularly the case with beat gestures that accompany intensifiers because beat gestures are aligned with the prosody of speech in a coordinated way. In other words, the production of a long vowel by the release of high-pressure air from the lungs is aligned with a beat gesture in a coordinated manner.

### 6.3. Communicating and Emphasizing the Degree of Something

Intensifiers are used to express the degree of something. They have an emphasizing function. In addition to being expressed by intensifiers, this emphasis can be made by a gesture because using two modes of communication to express one thing means that it is an important part of the meaning of the sentence. Here, a gesture is used to put more emphasis on one part of the meaning (the degree of something). In other words, gestures that are used with intensifiers in literal and metaphoric sentences can have a communicative emphasizing function. Speakers use these gestures to highlight a certain part of the meaning of the sentence. They are used to bring the size of something into the focus of attention and tell the addressee that this part of the meaning of the sentence is especially important. This is why it is expressed by both words and gestures in two modes of communication. Therefore, the use of gestures with intensifiers in literal and metaphoric sentences can be explained from a cognitive or a communicative perspective, although both of them can be in operation at the same time.

## 7. Conclusions and Suggestions for Future Research

Based on the results of this study, it can be concluded that the way that intensifiers are embodied in literal statements is similar to the way that they are embodied in metaphoric statements. When an intensifier is used with a literal or metaphoric sentence, there is a higher probability of using a gesture with that sentence. While using an intensifier with a literal sentence raises the probability of using an iconic or beat gesture, using an intensifier with a metaphoric sentence raises the probability of using a metaphoric or beat gesture. This could mean that intensifiers are embodied in terms of representational gestures (iconic and metaphoric) or beat gestures when they are used with literal or metaphoric sentences. Similar to our previous three studies [37,38,40], the results of this study support the key assumptions of the strong versions of the embodiment; that is, understanding literal and metaphorical statements involve basically similar embodiment processes.

Finally, it must be noted that, like any other study, this study had some limitations. Perhaps the most important limitation was the lack of accessibility to people from a variety of linguistic backgrounds. If the study had been conducted on several groups of participants with various mother tongues, more reliable results could have been obtained. Therefore, in future research projects, more comprehensive studies can be conducted on the use of gestures with intensifiers in various languages. Also, the data of this study were collected in a storytelling context. If the data had been collected in a more natural context, more reliable results could have been obtained. Therefore, in future research projects, the use of gestures with intensifiers can be examined in natural contexts by collecting data from daily interactions.

## Figures and Tables

**Table 1:** Number of literal sentences used with/without an intensifier and with iconic/beat gestures.

Literal sentences used with intensifiers	Literal sentences used with intensifiers and with iconic gestures	Literal sentences used with intensifiers and with beat gestures	Literal sentences used with intensifiers and without gesture
145	68 (46.8%)	47 (32.4%)	30 (20.6%)
Literal sentences used without intensifiers	Literal sentences used without intensifiers and with iconic gestures	Literal sentences used without intensifiers and with beat gestures	Literal sentences used without intensifiers and without gestures
260	67 (25.7%)	47 (18%)	146 (56.1%)

**Table 2:** Number of metaphors used with/without intensifier and with metaphoric/beat gestures.

Metaphors used with intensifiers	Metaphors used with intensifiers and with metaphoric gestures	Metaphors used with intensifiers and beat gestures	Metaphors used with intensifiers without gesture
156	80 (51.2%)	55 (35.2%)	21 (13.4%)
Metaphors used without intensifiers	Metaphors used without intensifiers and with metaphoric gestures	Metaphors used without intensifiers and with beat gestures	Metaphors used without intensifiers and without gesture
208	53 (25.4%)	49 (23.5%)	106 (50.9%)

## Data Availability

Data sharing is not applicable.

## Appendix A

**English translations of literal sentences used with intensifiers in the stories.**

1. The factory was really big.
2. It was very far away from their home.
3. The house was in a very wide area.
4. The village was very far from city center.
5. The conditions of life were too harsh.
6. The desert was too large.
7. This area was too high for him to reach the peak.
8. Her house was too small.
9. The boxes were so heavy that he could not move them.
10. The factory was so large that it was hard to walk from one side of the factory to other side.
11. The house was so big.
12. The distance between the city and the surrounding villages was so long.
13. He was living in really hard conditions.
14. There was a really deep gap between the poor and the rich.
15. The speed of the car was really fast.
16. She was walking really slowly.
17. The plain was rather large and open-ended.
18. He became rather strong.
19. The speed of movement was rather fast.
20. The walls were rather high and strong.
21. They took absolutely everything.
22. It was absolutely forbidden.
23. She had no choice but to be absolutely silent.
24. She absolutely supported her friends.

**English translations of metaphorical sentences used with intensifiers in the stories.**

1. He was fighting against a very high mountain of challenges.
2. The animosity was very deep and rooted.
3. He had very high ambitions for her future life.
4. Her kind behavior had a very deep effect on her friends.
5. The walls of distrust were too high between them.
6. The economic gap between people was too large.
7. The speed of changes was too high.
8. Her beliefs were too strong.
9. His beliefs were so strong that was impossible change them.
10. He was surrounded by so many challenges.
11. She had a so flexible behavior toward people.
12. The event had so many far-reaching consequences.
13. The advice was really a candle for him in those dark days.
14. He was swimming in a really stormy sea of challenges.
15. After that event, a really new chapter opened in her life.
16. The bond of love between her and her friends was really strong.
17. The difficulties seemed to be rather large and unconquerable.
18. It seemed to be a rather high ambition.
19. A large number of people were living at rather low levels of economic power.
20. Expectations were rather high.
21. His life could have been completely destroyed.
22. The road leading to his dreams was a completely one-way road for him.
23. The road to her dreams was a completely bright road.
24. She a completely pure heart that was full of kindness.
